# HIV-1 RNA May Decline More Slowly in Semen than in Blood following Initiation of Efavirenz-Based Antiretroviral Therapy

**DOI:** 10.1371/journal.pone.0043086

**Published:** 2012-08-13

**Authors:** Susan M. Graham, Sarah E. Holte, Joan A. Dragavon, Kelly M. Ramko, Kishor N. Mandaliya, R. Scott McClelland, Norbert M. Peshu, Eduard J. Sanders, John N. Krieger, Robert W. Coombs

**Affiliations:** 1 Department of Medicine, University of Washington, Seattle, Washington, United States of America; 2 Department of Global Health, University of Washington, Seattle, Washington, United States of America; 3 Department of Medical Microbiology, University of Nairobi, Nairobi, Kenya; 4 Centre for Geographic Medicine and Research – Coast, Kenya Medical Research Institute, Kilifi, Kenya; 5 Department of Biostatistics, University of Washington, Seattle, Washington, United States of America; 6 Department of Biostatistics and Biomathematics, Fred Hutchinson Cancer Research Center, Seattle, Washington, United States of America; 7 Department of Laboratory Medicine, University of Washington, Seattle, Washington, United States of America; 8 PathCare Kenya, Mombasa, Kenya; 9 Department of Epidemiology, University of Washington, Seattle, Washington, United States of America; 10 Nuffield Department of Clinical Medicine, University of Oxford, Headington, United Kingdom; 11 Department of Urology, University of Washington, Seattle, Washington, United States of America; 12 Department of Urology, VA Puget Sound Health Care System, Seattle, Washington, United States of America; Asociacion Civil Impacta Salud y Educacion, Peru

## Abstract

**Objectives:**

Antiretroviral therapy (ART) decreases HIV-1 RNA levels in semen and reduces sexual transmission from HIV-1-infected men. Our objective was to study the time course and magnitude of seminal HIV-1 RNA decay after initiation of efavirenz-based ART among 13 antiretroviral-naïve Kenyan men.

**Methods:**

HIV-1 RNA was quantified (lower limit of detection, 120 copies/mL) in blood and semen at baseline and over the first month of ART. Median log_10_ HIV-1 RNA was compared at each time-point using Wilcoxon Signed Rank tests. Perelson’s two-phase viral decay model and nonlinear random effects were used to compare decay rates in blood and semen.

**Results:**

Median baseline HIV-1 RNA was 4.40 log_10_ copies/mL in blood (range, 3.20–5.08 log_10_ copies/mL) and 3.69 log_10_ copies/mL in semen (range, <2.08–4.90 log_10_ copies/mL). The median reduction in HIV-1 RNA by day 28 was 1.90 log_10_ copies/mL in blood (range, 0.56–2.68 log_10_ copies/mL) and 1.36 log_10_ copies/mL in semen (range, 0–2.66 log_10_ copies/mL). ART led to a decrease from baseline by day 7 in blood and day 14 in semen (p = 0.005 and p = 0.006, respectively). The initial modeled decay rate was slower in semen than in blood (p = 0.06). There was no difference in second-phase decay rates between blood and semen.

**Conclusions:**

Efavirenz-based ART reduced HIV-1 RNA levels more slowly in semen than in blood. Although this difference was of borderline significance in this small study, our observations suggest that there is suboptimal suppression of seminal HIV-1 RNA for some men in the early weeks of treatment.

## Introduction

Effective antiretroviral therapy (ART) can greatly decrease HIV-1 transmission risk from treated individuals to their sexual partners [1]–[3]. These dramatic reductions in sexual transmission likely reflect decreased genital shedding of infectious HIV-1 [4]. While models suggest that widespread ART could help curb the global pandemic [5], it is important to understand how quickly, to what extent, and for how long different ART regimens suppress genital HIV-1 shedding in different populations.

Previously, we documented the decrease in genital HIV-1 shedding as women initiated World Health Organization (WHO)-recommended first-line therapy containing two nucleoside reverse transcriptase inhibitors (NRTI) plus a non-nucleoside reverse transcriptase inhibitor (NNRTI), primarily nevirapine [6]. Because transmission from men to women accounts for many infections in sub-Saharan Africa [7], the effect of NNRTI-based therapy on seminal HIV-1 RNA levels is likely a key determinant of ART’s preventive impact. We hypothesized that initiation of efavirenz-based ART would produce a rapid decrease in seminal HIV-1 RNA during the first month of therapy, but that the time course and magnitude of decay in semen might differ from events observed in blood.

## Methods

### Study Population and Procedures

HIV-1-seropositive Kenyan men attending a research clinic between July 2008 and July 2009 were invited to participate if they were eligible for ART according to Kenyan National Guidelines at the time (CD4 count ≤250 cells/µL or AIDS-defining illness) and were willing to undergo intensive follow-up including collection of semen. The initial regimen was stavudine or zidovudine combined with lamivudine in a single tablet twice daily plus efavirenz 600 mg once daily. Adherence to therapy was monitored by pill count, and directly administered ART was used to observe one of two daily doses at each visit.

Eligible men were screened for sexually transmitted infections (STI) before ART initiation. Study visits were conducted at baseline (day 0) then on days 2, 4, 7, 14, and 28 after ART initiation. At each visit, counseling staff interviewed participants using standardized questionnaires about recent sexual behavior, health status, and medication adherence. A standardized physical examination was performed, and urine was collected for STI screening. Blood was collected for CD4 cell counts on days 0 and 28, and for plasma HIV-1 RNA on days 0, 2, 7, 14 and 28. Participants collected semen in sterile urine specimen cups or non-reactive condoms (Durex® Avanti, SSL International, Anderson, SC, USA) according to a standard protocol [8], and submitted specimens within 2 hours of ejaculation.

This study was approved by ethical review committees at the Kenya Medical Research Institute and University of Washington (UW). All participants provided written informed consent.

### Laboratory Methods

STI screening included testing for urine leukocyte esterase (Multistix 10 SG, Bayer Diagnostics, Bridgend, UK), urethritis (>5 leukocytes per 100× field in Gram-stained urethral secretions), *Trichomonas vaginalis* (In-Pouch TV®, BioMed Diagnostics, White City, OR, USA), and *Chlamydia trachomatis* and *Neisseria gonorrhoeae* (Aptima GC/CT Detection System®, GenProbe Inc., San Diego, CA, USA). CD4 counts were determined using FACS Count (Becton Dickinson, Forest Lakes, NJ, USA). Semen was processed immediately after submission, following our published methods [8]. Specimens for HIV-1 RNA testing were frozen within 8 h of collection, then stored at −70°C until shipment to Seattle. HIV-1 RNA was quantified in the UW Retrovirology Laboratory using an independently validated real-time PCR amplification assay [9], [10]. The lower limit of quantification was 120 copies/mL.

### Data Analysis

HIV-1 RNA was log_10_-transformed, and set at ½ the lower limit of detection if the result was below this limit. Median log_10_ HIV-1 RNA values were compared across time-points using Wilcoxon Signed Rank tests. The Perelson model for two-phase viral decay was used to describe mean log_10_ blood and seminal HIV-1 RNA levels over time [11]. Nonlinear mixed effects methods with fixed effects for compartment (blood vs. semen) and random effects for baseline HIV-1 RNA and second-phase decay were used to compare decay rates [12]. Participants with undetectable seminal HIV-1 RNA at baseline were excluded from modeling of decay in semen. Otherwise, all measures were included until the first of two subsequent undetectable measures was achieved.

A minimum sample size of 13 men was required, based on a paired-sample t test comparing the HIV-1 RNA shedding end-points (baseline and day 28) with an anticipated difference of ≥1 log_10_ copies/mL, an estimated standard deviation of 1.0 log_10_ copies/mL, α = 0.05, 90% power, and a two-sided hypothesis test. Our target enrollment was 16 men, to allow for up to 20% loss to follow-up at day 28. Analyses were conducted using SPSS (version 15.0; IBM Corp., Armonk, NY, USA) and S-Plus (version 7.0; TIBCO Software, Inc., Palo Alto, CA). A two-sided p value <0.05 was considered significant.

## Results

### Study Participants

Thirteen men participated, with median age 32 years (inter-quartile range [IQR], 28–34 years) and median education 10 years (IQR, 6–12 years). Five men reported sex with women only, two reported sex with both women and men, and six reported sex with men only. Their median CD4 count was 204 cells/µL (IQR, 130–248 cells/µL). WHO stage was I for two, II for five, III for four, and IV for two men. All participants denied urethritis symptoms at baseline, although one *C. trachomatis* infection was diagnosed and treated. No genital ulcers were observed at baseline.

Eleven men completed the study through day 28, and only 3 of 78 (3.8%) expected visits were missed (one on day 7, two on day 28). One man failed to provide any semen specimens due to severe phimosis and balanitis. Median ART adherence was 100% (range, 96.7%–100%) for once-daily efavirenz tablets and 98.3% (range, 95.0%–100%) for twice-daily combined NRTI tablets. No new urethral infections or genital ulcers were observed.

### HIV-1 RNA Levels

Median baseline HIV-1 RNA was 4.40 log_10_ copies/mL in blood (range, 3.20–5.08 log_10_ copies/mL) and 3.69 log_10_ copies/mL in semen (range, <2.08–4.90 log_10_ copies/mL). The median reduction in HIV-1 RNA by day 28 was 1.90 log_10_ copies/mL in blood (range, 0.56–2.68 log_10_ copies/mL) and 1.36 log_10_ copies/mL in semen (range, 0–2.66 log_10_ copies/mL). Table 1 presents median HIV-1 RNA in blood and semen after ART initiation. The decrease was significant by day 7 in blood and day 14 in semen (p = 0.005 and p = 0.006, respectively). The decrease in seminal HIV-1 RNA by day 28 was not associated with genitourinary inflammation, symptoms, or reported adherence.

**Table 1:** Median Log_10_ HIV-1 RNA Level During the First Month of Antiretroviral Therapy among 13 Kenyan Men.

Time-point	Copies/mL in Blood Plasma (IQR)	Copies/mL in Seminal Plasma (IQR)
Baseline	4.40 (3.91–4.71)	3.69 (2.95–4.37)
Day 2*	3.81 (3.46–4.45), P = 0.08	3.43 (2.01–4.64), P = 0.66
Day 4*	–	3.23 (2.77–4.17), P = 0.37
Day 7* †	2.87 (2.35–2.98), P = 0.005	3.26 (2.47–4.01), P = 0.08
Day 14* †	2.74 (2.27–3.06), P = 0.003	2.45 (<2.08–3.53), P = 0.006
Day 28†	2.47 (2.31–2.68), P = 0.003	<2.08 (<2.08–3.21), P = 0.005

P values given are for paired comparisons to baseline levels for each sample type.

IQR  =  Inter-quartile range.

* Due to public holidays or other scheduling difficulties, eleven visits (15.9%) occurred one day before or one day after the scheduled visit date.

† One day 7 visit was missed and two day 28 visits were missed. All semen samples were missing for one participant. Blood samples were missing on day 7 (2 samples) and day 14 (1 sample).

HIV-1 RNA was detected in the blood of all men (100%) on day 0, 11 of 12 men (91.7%) on day 14, and 9 of 11 men (81.8%) on day 28. In contrast, HIV-1 RNA was detected in the semen of 10 of 12 men (83.3%) on day 0, 8 of 12 men (66.7%) on day 14, and 4 of 10 men (40.0%) on day 28. Detectable HIV-1 RNA levels on day 28 ranged from 206–1,240 copies/mL in blood and 120–5,128 copies/mL in semen, including three men with seminal HIV-1 RNA levels >1,000 copies/mL. Baseline seminal HIV-1 RNA was higher among the four men with detectable seminal HIV-1 RNA than the six men with undetectable seminal HIV-1 RNA at day 28, although this difference was not statistically significant (4.20 log_10_ copies/mL vs. 3.23 log_10_ copies/mL, p = 0.10).

### Modeling of HIV-1 RNA Decay Rates

**Figure 1:**
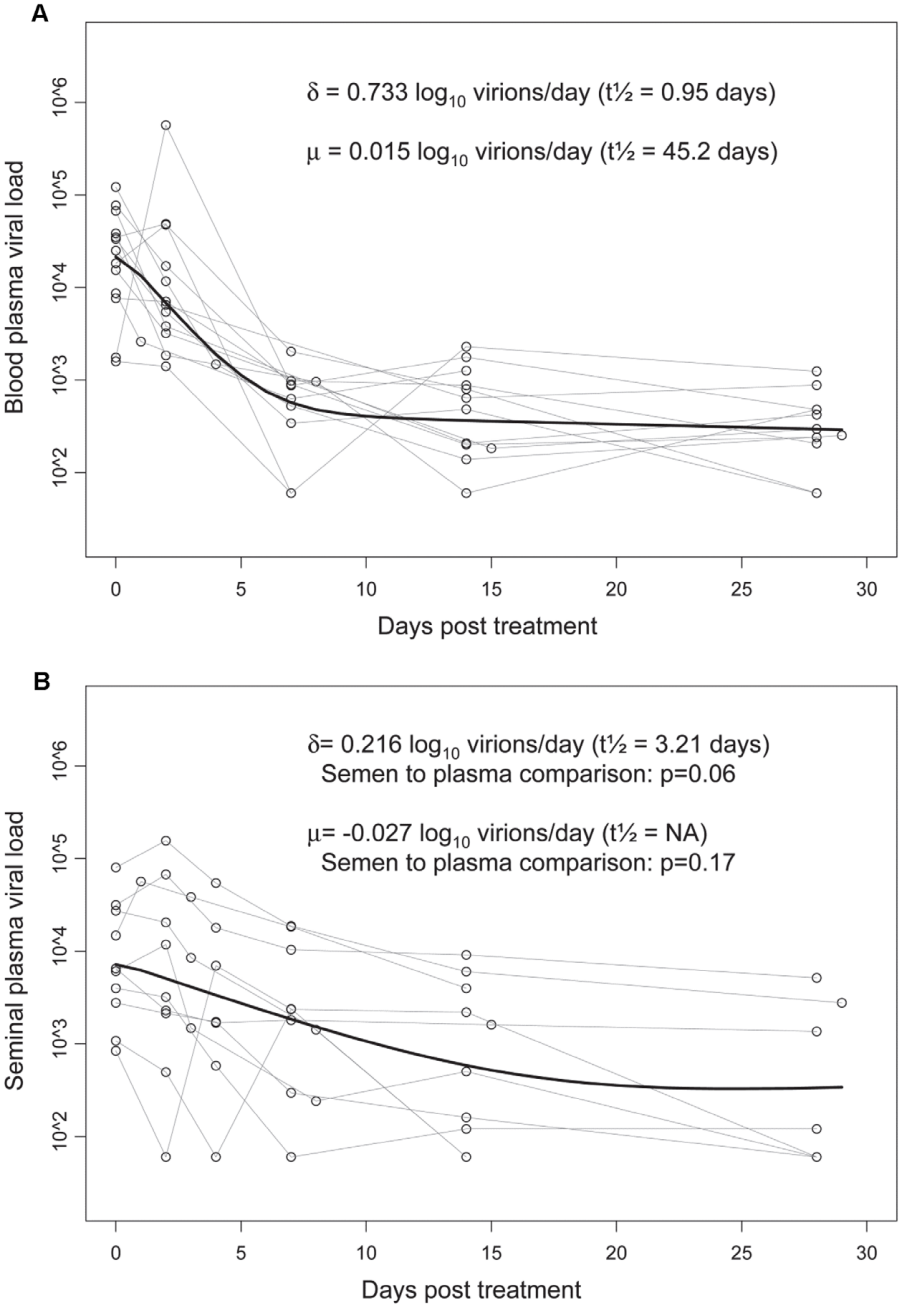
Decrease in HIV-1 RNA in blood and semen during the first month of antiretroviral therapy. Figure 1a presents results for blood plasma, and Figure 1b presents results for seminal plasma. Open, connected circles represent included data points (see methods). The bold line represents the modeled estimate. Delta (δ) is the estimate for the initial, rapid phase of decay, and mu (μ) the estimate for the subsequent, slower phase of decay. The half-life in days for each phase is equal to ln(2) divided by the decay rate in log_10_ virions/day. P values are presented for comparisons of the two decay rates in seminal plasma to those seen in blood plasma. NA = not applicable.

The Perelson model was used to evaluate HIV-1 RNA decay in blood (Figure 1a) and semen (Figure 1b) [11]. For each compartment, estimates were derived for both the initial rapid phase of decay (designated “δ”) and the subsequent, slower phase of decay (designated “μ”). For each estimate, a decay rate in log_10_ virions/day and a half-life are presented. Initial decay was significantly greater than zero in blood, but not in semen. Subsequent decay was not significantly greater than zero in either compartment. The initial modeled decay rate was slower in semen than in blood, although this difference was of borderline statistical significance (p = 0.06). There was no difference in the second phase of decay between semen and blood (p = 0.17).

## Discussion

The effect of ART on HIV-1 RNA levels in semen is poorly understood, but of particular importance for defining the infectious window following ART initiation. We conducted the largest study to date with frequent, longitudinal sampling of semen from antiretroviral-naïve Kenyan men during their first month of efavirenz-based ART. We found that first-phase decay was significantly greater than zero in men’s blood, but the initial decay rate in semen was not greater than zero and had an estimated half-life of over 3 days. Although the relatively small number of participants provided limited statistical power, this difference in initial decay rates between blood and semen was of borderline significance (p = 0.06). Thus, while we failed to demonstrate statistical significance in this small study, HIV-1 RNA decay during efavirenz-based therapy appears to be slower in semen than in plasma.

Prospective studies of men taking highly active ART have demonstrated reductions in seminal HIV-1 shedding [13]–[16], but also identified both persistent and intermittent seminal HIV-1 shedding during treatment [13]–[15]. Persistent or recurrent shedding might reflect poor antiretroviral penetration into male genital tract tissues and fluids, differences in pH between semen and blood, the presence of inhibitors in semen, or factors such as ejaculation frequency, which changes semen composition. In addition, decay after ART initiation may be affected by different distributions between blood and genital compartments of activated lymphocytes (responsible for the first phase of decay) versus long-lived infected cells such as memory T cells, macrophages, and dendritic cells (responsible for the second, slower phase of decay) [11], with a greater proportion of long-lived cells in the genital tract.

Only one published study evaluated seminal HIV-1 RNA decay [16]. In this study, seminal HIV-1 RNA level was assessed at multiple time points after therapy initiation for two patients: one ART-naïve man starting nevirapine-based therapy and one treatment-experienced man starting a salvage regimen. The estimated half-lives of seminal HIV-1 RNA were 1.1 days for first-phase decay and 12.1 days for second-phase decay [16]. Our estimate of initial decay was 2 days slower, and we did not find the second-phase decay to be significantly greater than zero. Our study has the advantage of having included only ART-naïve men with high initial viral loads and a standard regimen that differed only in the thymidine analog used (i.e., stavudine or zidovudine). Importantly, while zidovudine, stavudine, and lamivudine all achieve levels in semen at least as high as levels in blood [17], [18], efavirenz concentrations in semen have been estimated to be ≤10% of concentrations in blood [19]–[22].

Despite the somewhat slower estimated initial decay rate in semen, HIV-1 RNA levels were undetectable at a higher frequency in semen than in blood specimens (e.g., 60% vs. 18%) by day 28, in keeping with previous observations that seminal HIV-1 RNA is usually suppressed by efavirenz-containing triple-drug regimens [20]–[22]. However, break-through shedding and ongoing viral evolution have both been documented during efavirenz treatment [23], [24]. Continued seminal shedding among men taking ART has been related to higher baseline seminal HIV-1 RNA levels (important early in treatment) and suboptimal adherence (important throughout therapy) [14], [25]. Seminal HIV-1 RNA levels comparable to those we observed at day 28 in three men (>1,000 copies/mL) have been associated with heterosexual transmission events [4]. Of note, the only linked HIV-1 transmission event in the intervention group of the recently published trial of early ART for HIV-1 prevention was identified 3 months after the HIV-1-infected male partner initiated efavirenz-based treatment [3], [26]. Integrase inhibitors such as raltegravir lead to faster HIV-1 RNA clearance from blood plasma [27], and have been shown to penetrate seminal plasma well [28]. Studies are needed to compare the effect of efavirenz-containing versus raltegravir-containing regimens on genital and rectal HIV-1 shedding.

This is the first intensive study of seminal HIV-1 RNA levels among African men starting ART, and the WHO-recommended regimen used is the most common regimen worldwide [29], [30]. Participants reported excellent adherence, with ≥95% of doses taken. Given recent evidence documenting the efficacy of ART for HIV-1 prevention [3], our evaluation of efavirenz-based ART’s effect on HIV-1 RNA levels in semen is timely. However, our study had some important limitations. First, the small sample size provided limited power to detect potentially important clinical differences in decay rates between semen and blood. It proved difficult to recruit men due to intensive sampling, and we did not reach our target enrolment of 16 men. Second, it is possible that men had lower adherence than that estimated by pill counts. Because of small sample volumes, we did not measure ART levels in semen. Third, we used sensitive assays to detect small quantities of HIV-1 RNA in semen. The clinical importance of low-level shedding for transmission is unclear [4]. Finally, we did not measure cellular provirus or replication-competent virus, and so cannot comment on efavirenz-based ART’s impact on these outcomes.

In summary, HIV-1 RNA decay was somewhat slower in semen than blood among antiretroviral-naïve Kenyan men initiating efavirenz-based ART. Although most participants had undetectable HIV-1 RNA in semen by day 28, our results suggest that efavirenz-based ART alone might not optimally suppress seminal HIV-1 RNA for some men during the first several weeks after treatment initiation and that comparisons with other ART regimens are warranted.

## Acknowledgments

We would like to thank the research staff who helped make this project a success, the Kenya Medical Research Institute (KEMRI) and the International AIDS Vaccine Initiative (IAVI) for support of E. Sanders and use of clinical space, the Coast Provincial General Hospital for provision of laboratory space, and the KEMRI Director for permission to publish. Special thanks go to the men who participated in this study.
